# Effects of Tetrabasic Zinc Chloride on Growth Performance, Nutrient Digestibility and Fecal Microbial Community in Weaned Piglets

**DOI:** 10.3389/fvets.2022.905242

**Published:** 2022-06-16

**Authors:** Gang Zhang, Guoqing Hu, Zhenyan Yang, Jinbiao Zhao

**Affiliations:** ^1^State Key Laboratory of Animal Nutrition, College of Animal Science and Technology, China Agricultural University, Beijing, China; ^2^Nutrition Laboratory of Wellhope Foods Co., Ltd, Shengyang, China; ^3^Animal Husbandry and Fishery Science and Innovation Department, Jinan Institute of Agricultural Sciences, Jinan, China

**Keywords:** tetrabasic zinc chloride, zinc oxide, weaned piglets, growth performance, fecal microbiota

## Abstract

The study was conducted to explore the effects of tetrabasic zinc chloride (TBZC), as an alternative to zinc oxide (ZnO), on growth performance, serum indexes, and fecal microbiota of weaned piglets. A total of 108 weaned piglets (average initial body weight of 7.84 ± 0.97 kg) were randomly allocated into one of three dietary treatments with six replicate pens and six piglets per pen. The dietary treatments included a control diet (CON, negative control), a ZnO diet (CON + 1,600 mg Zn/kg from ZnO, positive control), and a TBZC diet (CON + 1,000 mg Zn/kg from TBZC). The average daily gain of pigs in the TBZC group was greater (*P* < 0.05) than those in CON and ZnO groups during the whole period. Piglets fed the ZnO and TBZC diets showed lower (*P* < 0.05) diarrhea incidence than those fed the CON diet during d 1-14 and the whole period. Piglets fed the TBZC diet had higher (*P* < 0.05) digestibility of crude protein and gross energy than those fed the CON diet. Serum concentrations of IGF-I and GH, as well as ALP activity, were significantly elevated (*P* < 0.05) in the TBZC treatment group compared to the CON group on d 14. Piglets fed the ZnO diet had greater (*P* < 0.05) acetate and total short-chain fatty acids concentrations, while the TBZC diet had greater (*P* < 0.05) fecal acetate and propionate concentrations on d 28. Moreover, TBZC supplementation significantly increased (*P* < 0.05) microbial α-diversity compared with the CON group. The fecal microbiota of piglets in ZnO and TBZC treatment groups tended (*P* = 0.08) to have greater relative abundance of Prevotellaceae compared with the CON piglets. In conclusion, TBZC acted as a suitable alternative to ZnO to reduce zinc excretion, and improve growth performance of weaned piglets.

## Introduction

Early weaning of piglets is a key technology for intensive pig production. It can not only improve the productivity of sows and the utilization efficiency of pens, reduce the risk of maternal transmission of diseases, but also improve piglet growth performance (1). The digestive system of early-weaned piglets is immature and has no ability to utilize plant-based solid feed efficiently. In addition, piglets are subjected to psychological, nutritional, and environmental stresses after weaning, which often leads to piglet diarrhea, low feed intake and increased mortality, causing huge losses in the pig industry (2). Over the past decades, pharmacological doses of more than 2,000 mg/kg zinc oxide (ZnO) have been widely used in weaned piglet diets to alleviate post-weaning diarrhea and improve growth performance (3, 4). However, the negative effects of long-term high-dose use of ZnO cannot be ignored. Generally, about 10.0 to 25.0% of dietary ZnO is absorbed by pig's intestine, while most of the Zn is excreted through feces, causing environmental pollution, waste of Zn source, and microbial resistance development (4, 5). Moreover, excessive output of Zn from pigs' feces to the water and soil produces toxicity to crops and threats animal and human health (3, 6). Given the long-term impact of Zn excretions on the environment, considerable efforts have been made to increase the bioavailability of Zn, including organic Zn, nanosized ZnO and coated ZnO (7). However, the limitations of the application of these novel Zn sources in feed industry are complex processing technologies and high production cost (8). Recently, tetrabasic zinc chloride (TBZC), which is far cheaper than organic Zn, was launched as a suitable replacement to ZnO (8).

Tetrabasic zinc chloride is an inorganic Zn using the crystallization process. Compared with inorganic Zn such as ZnO, TBZC contains less heavy metal contamination, and has better palatability, as well as higher relative bioavailability. Diet supplemented with TBZC could inhibit oxidation of dietary nutrients during storage duration due to the high stable chemical property and low hygroscopic capacity of TBZC (6). Dietary TBZC supplementation at 1,000 to 1,250 mg Zn/kg could achieve the same anti-diarrhea and growth-promoting effects as pharmacological doses of ZnO, with lower fecal Zn excretion (6). A publication reported that TBZC was more effective than ZnO in upregulating the expression of intestinal tight junction proteins, and decreasing the permeability of the intestinal epithelium and the fecal scores (9). Therefore, as a Zn source with high bioavailability, TBZC can reduce the fecal output of Zn effectively while controlling the diarrhea incidence of piglets, and is expected to become a promising alternative for high-dose ZnO.

The objective of this experiment was to explore effects of TBZC, as an alternative to ZnO, on growth performance, nutrient digestibility, Zn excretion and fecal microbial composition of weaned piglets.

## Materials and Methods

All protocols mentioned were reviewed and approved by the Institutional Animal Care and Use Committee of China Agricultural University (Beijing, China; CAU NO. AW01202213-1). The experiment was carried out at Swine Research Unit of China Agricultural University (Hebei, China).

### Experimental Products

The Zn purity of TBZC [Zn_5_(OH)_8_Cl_2_·H_2_O] is 55%, which was supplied by Changsha Xingjia Biotechnology Company (Changsha, China), and the ZnO was a common feed-graded source and provided by Swine Research Unit of China Agricultural University. The TBZC supplementation content (1000 mg Zn/kg) were determined according to the previous study (6), and the Chinese ministry of agriculture stipulates that the maximum allowed level of ZnO for weaned piglets is 1,600 mg Zn/kg.

### Animals, Dietary Treatments and Experimental Design

A total of 108 healthy weaned piglets (Duroc × Landrace × Yorkshire, average initial body weight of 7.84 ± 0.97 kg) were randomly allocated to one of three dietary treatments according to their body weight. Each treatment comprised 6 replicate pens, with 6 pigs (3 barrows and 3 gilts) per pen. The dietary treatments included: a corn-soybean meal control diet without any Zn source addition (CON, negative control), a ZnO diet (CON + 1,600 mg Zn/kg from ZnO, positive control), and a TBZC diet (CON + 1,000 mg Zn/kg from TBZC). All diets were formulated to meet or exceed the nutrient requirements, except for Zn, of weaned piglets recommended by National Research Council (10), and were presented in mash form (Table 1). The experiment lasted for 28 days and was divided into two phases: d 1 to 14 and d 15 to 28. Chromic oxide (3 g/kg) was added to the Phase 2 diet to determine apparent total tract digestibility (ATTD) of energy and nutrients.

**Table 1:** Composition and nutrient levels of the basal diets (%, as-fed basis).

**Ingredients**	**D 1 to 14**	**D 15 to 28**
Corn	51.64	58.53
Soybean meal, 43%	16.85	13.40
Extruded soybean	14.20	11.00
Fish meal, 64.6%	4.00	5.00
Whey powder, 3.8%	8.00	7.00
Soybean oil	1.65	1.32
Dicalcium phosphate	1.21	1.10
Limestone	0.74	0.50
Salt	0.30	0.50
L-lysine HCl, 98%	0.46	0.40
DL-Methionine, 98%	0.12	0.15
L-Threonine, 98%	0.13	0.10
Choline chloride, 40% choline	0.20	0.20
Chromic oxide	–	0.30
Vitamin-mineral premix^a^	0.50	0.50
**Calculated nutrient levels, %**		
Digestible energy, kcal/kg	3,550	3,480
Calcium	0.85	0.75
Available P	0.46	0.44
Standardized ileal digestible lysine	1.41	1.30
Standardized ileal digestible methionine	0.41	0.40
Standardized ileal digestible threonine	0.80	0.74
Standardized ileal digestible tryptophan	0.20	0.21
**Analyzed nutrient levels, %**		
Dry matter	88.87	88.40
Crude protein	20.32	18.78
Neutral detergent fiber	10.72	11.74
Acid detergent fiber	4.29	4.42
Ether extract	4.62	3.04
Ash	5.64	5.81

a *Vitamin and mineral premix provided the following per kilogram of diet: 12,000 IU vitamin A as vitamin A acetate, 2,500 IU vitamin D as vitamin D_3_, 30 IU vitamin E as DL-α-tocopheryl acetate, 12 μg of vitamin B_12_, 3 mg vitamin K as menadione sodium bisulfate, 15 mg D-pantothenic acid as calcium pantothenate, 40 mg of nicotinic acid, 400 mg choline as choline chloride, 30 mg Mn as manganese oxide, 90 mg Fe as iron sulfate, 10 mg Cu as copper sulfate, 0.35 mg I as ethylenediamine dihydroiodide, and 0.3 mg Se as sodium selenite*.

All piglets were fed in 1.5 × 1.2 m^2^ pens with plastic slatted floors in a room where the feeding environment can be controlled automatically. The initial room temperature was maintained at 28°C and then gradually decreased by 1°C per week, and the relative humidity was controlled at 60% to 70%. Each pen was provided with a stainless steel feeder and a nipple drinker to allow the piglet *ad libitum* access to feed and water in the whole experiment. The pens and barn were cleaned every day to keep the environment sanitary.

### Sample Collection

Individual piglet weight and feed consumption from each pen were recorded on d 0, 14, and 28 to determine average daily gain (ADG), average daily feed intake (ADFI) and feed to gain ratio (F: G). The clinical signs of diarrhea were assessed every morning and afternoon by technicians blinded to dietary treatments, and the visual observations were carried out for each piglet, with touching the butt of each piglet to assist a better assessment. The below equation was used to calculate diarrhea incidence of weaned piglets:


(1) $$\begin{array}{l} \text{Diarrhea incidence }(\%)\;=\;(\text{number of piglets with diarrhea}\\ \times \text{ diarrhea days})/(\text{total number of piglets}\\ \times \text{ total observational days})\;\times \;100 \end{array}$$

Representative feed samples (500 g) of each treatment were collected before the start of the experiment. On d 27, ~300 g of fresh fecal samples were collected from each pen and stored under the condition of −20°C. After thawing, fecal samples from the same pen were pooled and mixed thoroughly, and then oven-dried at 65°C for 72 h. All feed and fecal samples were finely grounded to pass through a 1 mm sieve (40 mesh) before analysis. Fresh sample of the feces from each pen (1 piglet per pen) were collected via rectal massage and immediately snap-frozen in liquid nitrogen, and stored at −80°C for the determination of microbial composition and short-chain fatty acids (SCFA).

On d 14 and d 28, one barrow around average body weight from each pen was selected to collect blood samples from the precaval vein using vacuum tubes. The blood sample was rested for 45 min at room temperature prior to plasma separation. Serum was collected after centrifugation at 3,000 × g for 15 min at 4°C, and stored at −20°C for the further analysis.

### Analytical Methods

Feed and fecal samples were analyzed for dry matter (DM; method 930.15), crude protein (CP; method 984.13), ether extract (EE; method 920.39), and ash (method 942.05) in accordance with AOAC procedures (11). Gross energy (GE) was measured by an automatic adiabatic oxygen bomb calorimeter (Parr 6400, Calorimeter, Moline, IL, USA). Feed samples were also analyzed for neutral detergent fiber (NDF) and acid detergent fiber (ADF) using fiber filter bags and fiber analyzer equipment (Ankom Technology, Macedon, NY, USA) according to the methodology of van Soest et al. (12). The concentrations of chromium and Zn were measured using atomic absorption spectroscopy (PinAAcle 900F; Perkins Elmer, USA) after the samples were wet-digested with a mixture of nitric acid and perchloric acid (3:1). Nutrient digestibility was calculated by the following equation:


(2) $$\begin{array}{l} ATTD\;\left(\%\right)\;=\;100\;-\;\left(Crd\;\times \;Nf\right)\;/\;\left(Crf\;\times \;Nd\right)\;\times \;100 \end{array}$$

where Crd represents the chromium concentration in the diet (g/kg), Nf is the nutrient level in feces (g/kg), Crf is the chromium concentration in feces (g/kg), and Nd is the nutrient level in the diet (g/kg).

Serum insulin-like growth factor-I (IGF-I) and growth hormone (GH) were determined using the assay kits (Beijing Sino-UK Institute of Biological Technology, Beijing, China). The alkaline phosphatase (ALP), aspartate aminotransferase (AST), and alanine aminotransferase (ALT) activities were measured by assay kits (Beijing Sino-UK Institute of Biological Technology, Beijing, China) using an automatic bio-chemical analyzer (Hitachi 7020, Hitachi High-Technologies Corporation, Japan).

The SCFA concentrations in feces were determined following a modified method (13). Briefly, frozen samples were thawed at room temperature, about 0.5 g of fecal sample was weighed and diluted with 8 ml ultrapure water. After 30 min of sonication, the fecal sample solution was centrifuged at 5,000 × g for 10 min. The supernatant was diluted 50-fold in ultrapure water and then filtered through a 0.20 mm membrane filter for further determination.

Total microbial genomic DNA was extracted from the fecal samples using DNA Extraction Kit (Omega, USA). The V3–V4 region of 16S rRNA gene was amplified using the specific primers 338F and 806R. Barcoded amplicon libraries were separated by agarose gel electrophoresis and purified. Purified amplicons were pooled in equimolar amounts, and then sequenced using Illumina MiSeq platform (San Diego, US). Only sequences that overlap longer than 10 bp were merged. The sequences were clustered into operational taxonomic units (OTUs) at a 97% similarity, and chimeric sequences were removed using UCHIME algorithm. The Ribosomal Database Project (RDP) classifier was utilized to classify and annotate each OTU.

### Statistical Analysis

Raw data were checked for normality and outliers using the UNIVARIATE procedure of SAS 9.4 (SAS Institute Inc., Cary, NC, USA). The diarrhea incidence was compared with a chi-squared test. Other data were then analyzed using the GLMMIX procedures, and the Tukey HSD's test was used to adjust for multiple comparisons. The statistical model included the fixed effect of dietary treatment, body weight was included as a random effect. Pen served as the experimental unit for the growth performance and nutrient digestibility data, whereas individual piglet was used as the experimental unit for the other parameters. For the microbiome sequencing data, relative abundance of microbiota was compared with Kruskal-Wallis test for comparison of multiple groups. The linear discriminant analysis (LDA) effect size (LEfSe) analysis was conducted to determine the differential abundant taxonomies among different treatments, and LDA scores more than 2.0 were considered as having different abundance. Statistical significance was declared at *P* < 0.05, and a tendency for significance was considered when 0.05 ≤ *P* < 0.10.

## Results

### Piglet Performance and Diarrhea

The performance and diarrhea of piglets are shown in Table 2. The final BW was significantly greater (*P* < 0.05) in the TBZC treatment group compared with CON and ZnO treatment groups. The ADG in the TBZC group was only higher (*P* < 0.05) than that in the CON group during phase 2 (d 15 to 28), while higher (*P* < 0.05) than that in CON and ZnO groups during the overall 28-day period. However, ADFI and feed conversion ratio were not influenced (*P* > 0.10) by dietary treatments throughout the experiment. In addition, piglets offered the ZnO and TBZC diets exhibited lower (*P* < 0.05) diarrhea incidence than those fed the CON diet during phase 1 (d 1 to 14) and the overall experimental period.

**Table 2:** Effects of tetrabasic zinc chloride on growth performance and diarrhea incidence in weaned piglets.

**Item**	**CON**	**ZnO**	**TBZC**	**SEM**	***P*-Value**
Initial BW, kg	7.85	7.84	7.83	0.01	0.99
Final BW, kg	19.23^b^	19.50^b^	20.18^a^	0.22	0.03
**D 1 to 14**					
ADG, g	323	342	360	11.52	0.19
ADFI, g	515	523	557	14.74	0.15
Feed: Gain	1.57	1.53	1.55	0.02	0.31
Diarrhea incidence	16.07^a^	9.05^b^	8.04^b^		<0.01
**D 15 to 28**					
ADG, g	484^b^	493^ab^	523^a^	9.53	0.04
ADFI, g	913	909	941	23.99	0.61
Feed: Gain	1.89	1.83	1.80	0.04	0.27
Diarrhea incidence	7.59	6.40	4.46		0.15
**D 1 to 28**					
ADG, g	406^b^	417^b^	441^a^	7.01	0.01
ADFI, g	714	716	749	15.09	0.23
Feed: Gain	1.73	1.68	1.67	0.02	0.21
Diarrhea incidence	11.83^a^	7.77^b^	6.25^b^		<0.01

a,b *Means within rows with different superscripts differed significantly (P < 0.05; n = 6)*.

*CON, a control diet without any Zn source addition; ZnO, CON + 1,600 mg Zn/kg from zinc oxide; TBZC, CON + 1,000 mg Zn/kg from tetrabasic zinc chloride; BW, body weight; ADG, average daily gain; ADFI, average daily feed intake*.

### Nutrient Digestibility and Fecal Zinc Concentration

Weaned piglets fed the TBZC diet had greater (*P* < 0.05) ATTD of CP and GE, and tended to increase (*P* < 0.10) the ATTD of DM and OM than those fed the CON diet (Table 3). Furthermore, the analyzed values of Zn in the CON, ZnO and TBZC diets were 54, 1,643, and 1,062 mg/kg, respectively (data not shown). The TBZC group showed lower fecal Zn concentration (*P* < 0.01) than the ZnO group, but greater (*P* < 0.01) than the CON group.

**Table 3:** Effects of tetrabasic zinc chloride on apparent total tract digestibility of nutrients and fecal zinc concentration in weaned piglets (%).

**Item**	**CON**	**ZnO**	**TBZC**	**SEM**	***P*-Value**
**Apparent nutrient digestibility**					
Gross energy	80.87^b^	81.59^ab^	82.72^a^	0.48	0.05
Dry matter	81.83	82.36	83.28	0.43	0.09
Organic matter	83.80	84.36	85.22	0.43	0.09
Crude protein	73.60^b^	75.18^ab^	77.88^a^	0.92	0.02
Ether extract	45.25	43.13	48.39	1.64	0.16
**Feces**					
Zinc, g/kg DM	0.35^c^	10.18^a^	7.32^b^	0.26	<0.01

a−c *Means within rows with different superscripts differed significantly (P < 0.05; n = 6). Organic matter was calculated as the difference between dry matter and ash*.

*CON, a control diet without any Zn source addition; ZnO, CON + 1,600 mg Zn/kg from zinc oxide; TBZC, CON + 1,000 mg Zn/kg from tetrabasic zinc chloride*.

### Serum Enzyme Activity and Growth-Related Hormone

On day 14, the concentrations of serum IGF-I and GH were significantly increased (*P* < 0.05) in the TBZC group compared with the CON group (Table 4). Piglets fed ZnO and TBZC diets exhibited greater (*P* < 0.05) ALP activity and lower ALT activity in serum than those fed the CON diets. On day 28, the serum IGF-I concentration was remarkably increased (*P* < 0.05) in the TBZC group compared with other groups. Moreover, serum ALP of the TBZC group and the ZnO group had a trend of increasing (*P* = 0.07).

**Table 4:** Effects of tetrabasic zinc chloride on serum enzyme activities and hormone levels in weaned piglets.

**Item**	**CON**	**ZnO**	**TBZC**	**SEM**	***P*-Value**
**D 14**					
ALT, U/L	50.00^a^	39.25^b^	34.25^b^	1.68	<0.01
AST, U/L	46.25	36.00	32.25	4.64	0.15
ALP, U/L	182.31^b^	269.19^a^	259.20^a^	22.81	0.04
GH, ng/mL	5.45^b^	6.06^ab^	6.85^a^	0.28	0.02
IGF-I, ng/mL	186.62^b^	205.82^b^	252.72^a^	7.44	<0.01
**D 28**					
ALT, U/L	63.67	66.83	66.17	5.78	0.92
AST, U/L	55.67	55.17	54.17	7.83	0.75
ALP, U/L	177.64	244.02	262.99	25.41	0.07
GH, ng/mL	6.45	6.67	7.33	0.34	0.19
IGF-I, ng/mL	239.28^b^	245.47^b^	276.17^a^	9.64	0.03

a,b *Means within rows with different superscripts differed significantly (P < 0.05; n = 6)*.

*CON, a control diet without any Zn source addition; ZnO, CON + 1,600 mg Zn/kg from zinc oxide; TBZC, CON + 1,000 mg Zn/kg from tetrabasic zinc chloride; ALT, alanine aminotransferase; AST, aspartate aminotransferase; ALP, alkaline phosphatase; GH, Growth hormone; IGF-I, Insulin-like growth factor-I*.

### Fecal Short-Chain Fatty Acids Concentrations

On day 14, piglets fed the ZnO diet showed a tendency (*P* = 0.06) to decrease lactic acid concentration in feces compared with piglets fed the CON diet (Table 5). On day 28, compared with the CON group, piglets fed the ZnO diet had higher (*P* < 0.05) acetate and total SCFA concentrations, while the TBZC diet had greater (*P* < 0.05) acetate, propionate and total SCFA concentrations in the fecal samples.

**Table 5:** Effects of tetrabasic zinc chloride on fecal short-chain fatty acids concentrations (mg/g) in weaned piglets.

**Item**	**CON**	**ZnO**	**TBZC**	**SEM**	***P*-Value**
**D 14**					
Lactic acid	1.05	0.34	0.75	0.20	0.06
Acetate	3.70	3.50	3.74	0.26	0.78
Propionate	1.70	2.19	2.03	0.16	0.11
Butyrate	1.01	1.44	1.34	0.17	0.19
Valerate	0.21	0.47	0.33	0.08	0.11
Total SCFA	7.91	8.34	8.41	0.63	0.79
**D 28**					
Lactic acid	0.30	0.62	0.52	0.19	0.49
Acetate	3.00^b^	3.50^a^	3.41^a^	0.12	0.02
Propionate	1.73^b^	2.00^ab^	2.24^a^	0.13	0.04
Butyrate	0.95	1.30	1.53	0.20	0.15
Valerate	0.25	0.34	0.43	0.08	0.33
Total SCFA	6.45^b^	7.97^a^	8.39^a^	0.38	0.01

a,b *Means within rows with different superscripts differed significantly (P < 0.05; n = 6)*.

*CON, a control diet without any Zn source addition; ZnO, CON + 1,600 mg Zn/kg from zinc oxide; TBZC, CON + 1,000 mg Zn/kg from tetrabasic zinc chloride; SCFA, short-chain fatty acids*.

### Microbiota Community

**Figure 1:**
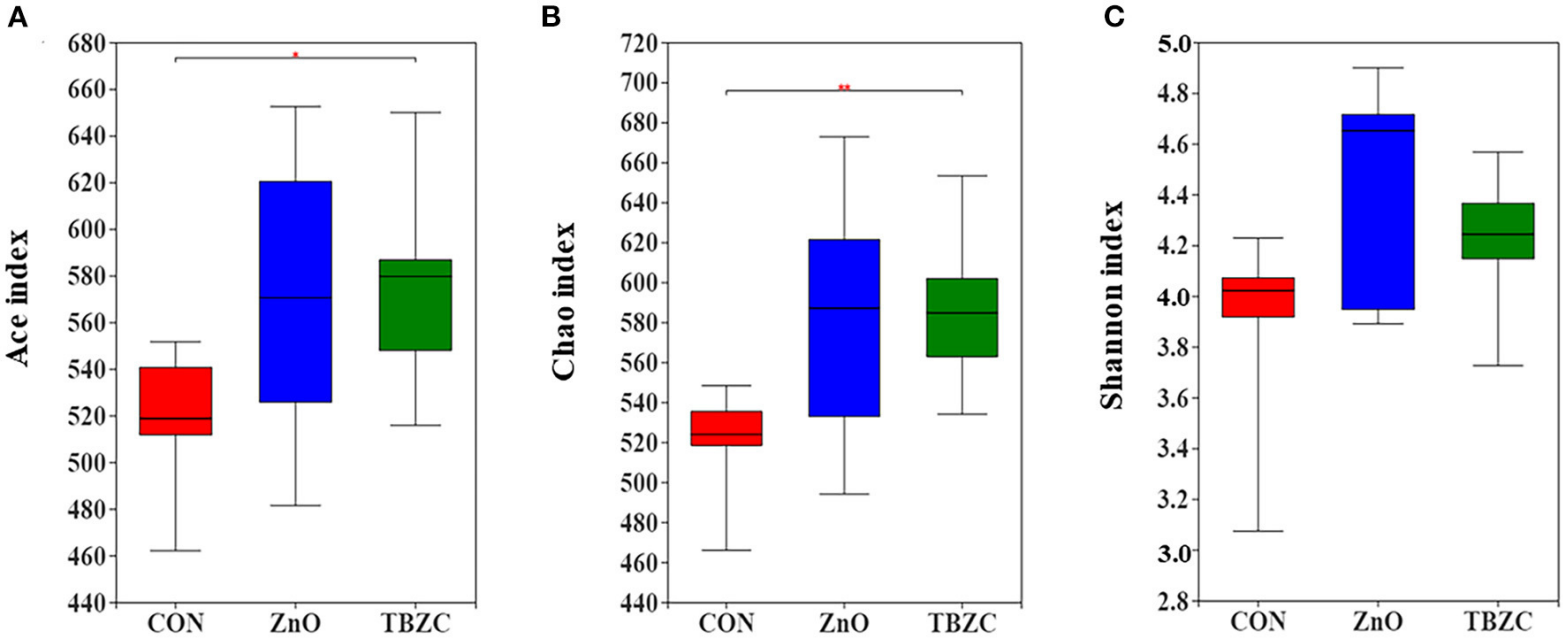
Microbial α-diversity in the fresh fecal samples of piglets. Ace **(A)**, Chao **(B)**, and Shannon **(C)** indices of the microbial community in different treatment groups. Data are the means of the indexes within each treatment (*n* = 6). CON, a control diet without any Zn source addition; ZnO, CON + 1,600 mg Zn/kg from zinc oxide; TBZC, CON + 1,000 mg Zn/kg from tetrabasic zinc chloride. *Represents 0.05 < *p* < 0.01, **represents *p* < 0.01.

**Figure 2:**
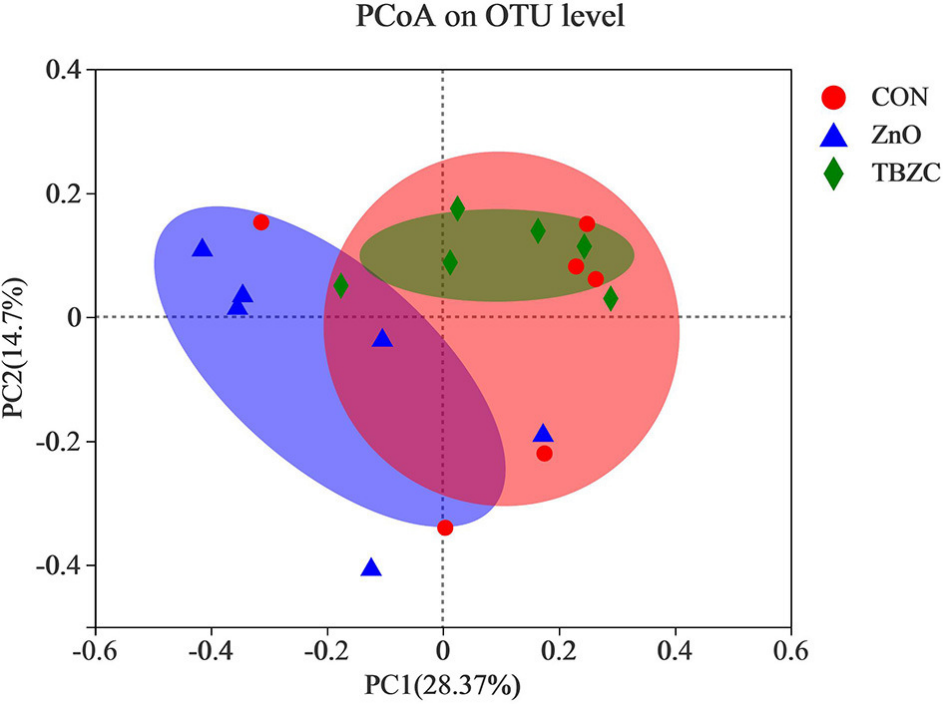
Principal coordinate analysis (PCoA) of the community structure in the fecal samples based on unweighted UniFrac distances (*n* = 6). CON, a control diet without any Zn source addition; ZnO, CON + 1,600 mg Zn/kg from zinc oxide; TBZC, CON + 1,000 mg Zn/kg from tetrabasic zinc chloride.

The microbial community richness and diversity for fecal samples was estimated by the Ace, Chao and Shannon indices (Figure 1). Alpha diversity analysis showed that dietary TBZC supplementation significantly increased (*P* < 0.05) the Ace and Chao indices of fecal microbiota compared with the CON group. The principal coordinates analysis (PCoA) plot based on the unweighted UniFrac distances showed that the microbiota composition in the ZnO group was significantly different from that of the TBZC group, but no distinct difference (*P* > 0.05) was detected in the microbial composition between the CON group and the ZnO or TBZC group (Figure 2).

**Figure 3:**
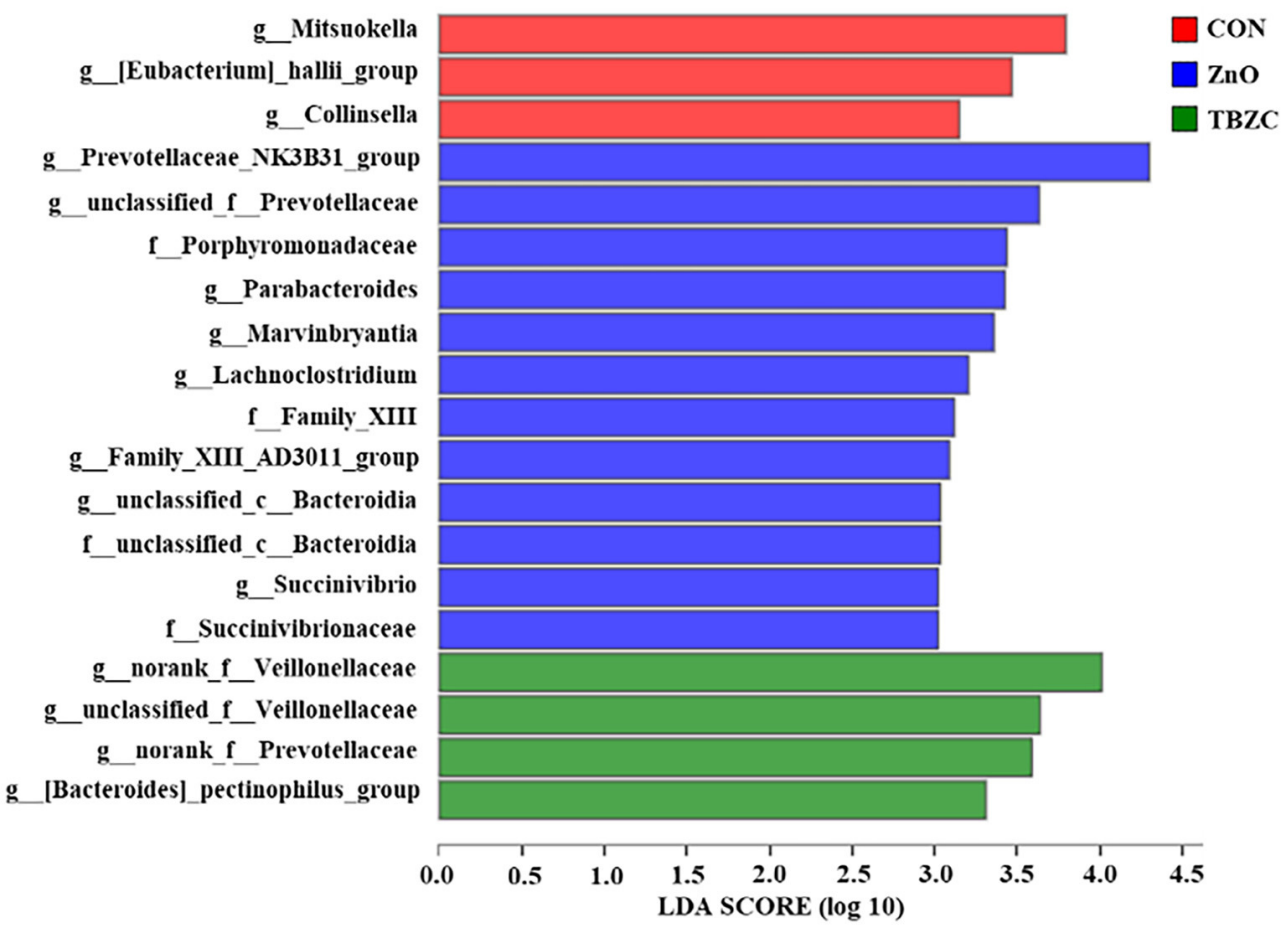
Linear discriminant analysis effect size (LEfSe) analysis of the fecal microbiota of weaned piglets (*n* = 6). The linear discriminant analysis (LDA) score chart is shown for species with an LDA score >2. Red, blue, and green squares represent the CON, ZnO, and TBZC groups, respectively. CON, a basal diet without any Zn source addition; ZnO, CON + 1,600 mg Zn/kg from zinc oxide; TBZC, CON + 1,000 mg Zn/kg from tetrabasic zinc chloride.

The relative abundance of the fecal microbial communities at the family level is shown in Table 6. The dominant taxa across all treatments were Prevotellaceae, Ruminococcaceae, Veillonellaceae, Lachnospiraceae, and Lactobacillaceae; these groups accounted for about 80% of the bacterial sequences. The fecal microbiota of piglets in ZnO and TBZC treatment groups tended (*P* = 0.08) to have greater relative abundance of Prevotellaceae compared with the CON piglets, whereas the relative abundance of Veillonellaceae tended to be lower (*P* = 0.09) in piglets fed ZnO than in those fed TBZC. The LEfSe was further used to identify the microbial markers with statistical difference among different treatments (Figure 3). The present results suggested that the relative abundance of 12 taxa of bacteria, such as *Prevotellaceae_NK3B31_group, Parabacteroides*, and *Succinivibrio*, in the ZnO treatment group was higher compared with the other treatment groups. Genera with higher abundance in the CON group included *Mitsuokella, Eubacterium_hallii_group* and *Collinsella*, whereas *g__norank_f__Veillonellaceae, g__unclassified_f__Veillonellaceae, g__norank_f__Prevotellaceae* and *Bacteroides_pectinophilus_group* were more abundant in the TBZC group.

**Table 6:** Effects of tetrabasic zinc chloride supplementation on relative abundance of fecal microbial community at family level in weaned piglets.

**Item**	**CON**	**ZnO**	**TBZC**	**SEM**	***P*-Value**
Prevotellaceae	23.92	30.17	31.27	2.36	0.08
Ruminococcaceae	15.09	18.98	11.73	3.10	0.32
Veillonellaceae	16.65	5.50	22.06	4.92	0.09
Lachnospiraceae	12.05	12.08	10.27	1.75	0.71
Lactobacillaceae	16.28	12.34	5.44	5.36	0.38
Bacteroidales_S24-7_group	4.33	5.75	2.92	1.98	0.62
Streptococcaceae	2.42	2.51	5.79	1.37	0.19
Acidaminococcaceae	1.74	1.20	2.18	0.35	0.21
Rikenellaceae	1.35	1.61	1.84	0.46	0.74
Clostridiaceae_1	0.46	1.31	0.69	0.33	0.22

*Data only demonstrated the top 10 bacteria at family level (n = 6)*.

*CON, a control diet without any Zn source addition; ZnO, CON + 1,600 mg Zn/kg from zinc oxide; TBZC, CON + 1,000 mg Zn/kg from tetrabasic zinc chloride*.

## Discussion

Zinc is an essential trace element to maintain various physiological functions of the body, which can help in the development of the gastrointestinal tract, modulating the immune response and antioxidant system, and thus affecting the growth and health of piglets (14–16). Numerous studies have demonstrated that supplementing weaned piglet diets with pharmacological doses of ZnO could alleviate weaning stress and improve growth performance (3, 17, 18). However, the results of this study showed that dietary supplementation of 1600 mg Zn/kg from ZnO failed to improve the growth performance of piglets, which was consistent with the results reported by Mavromichalis et al. (19). Schell and Kornegay (20) also reported that neither organic nor inorganic Zn could improve the growth performance of piglets at doses of 1,000 to 2,000 mg Zn/kg. As reported previously, doses below 2000 mg Zn/kg from ZnO is often unable to reach the effective growth promotion under conditions without other additives (21–23). Mavromichalis et al. (19) reported that Zn level of 750 mg/kg as TBZC did not enhance growth performance, and Zn level >1,500 mg/kg was no more efficacious on growth performance than the 1,500 mg/kg in weaned pigs. In addition, our previous study also found that dietary TBZC supplementation at 1,000 to 1,250 mg Zn/kg showed the same growth rate and lower fecal Zn excretion than pharmacological doses of ZnO (6). Based on these findings, we considered that the optimal dose for dietary TBZC supplementation to improve growth performance and reduce Zn excretion could be 1,000 mg Zn/kg. Moreover, as expected, the growth performance of piglets fed the TBZC diet was greater than those fed ZnO and CON diets. A previous study also reported that TBZC at 1,500 mg Zn/kg exhibited a growth-promoting effect comparable to that obtained by pharmacological doses of ZnO, and had better feed conversion efficiency (19). These results suggested that TBZC has great potential to be used as an alternative to conventional ZnO. However, the exact mechanisms of the growth-promoting effect by TBZC are still not fully elucidated. The positive effect of TBZC on growth performance of piglets is partly attributed to improved intestinal morphology and nutrient absorption (24). The improvement of metabolism, immunity and antioxidant status by TBZC may provide benefits to enhance the growth performance of piglets (6). TBZC has been shown to reduce intestinal permeability and improve mucosal barrier function (9). Dietary TBZC supplementation also involved in stimulating the secretion of ghrelin and growth hormone, thereby promoting muscle protein synthesis and cell proliferation (25).

Post-weaning diarrhea (PWD) is one of the most common causes of stunted growth and mortality in piglets, causing huge economic losses to the pig industry. In practice, ZnO at pharmacological doses (2,000 mg Zn/kg or more) is widely applied to weaned piglet diets to prevent occurrences of PWD (3, 26). In our study, dietary TBZC supplementation significantly reduced the diarrhea incidence in piglets, and its effect was comparable to that of the ZnO treatment group, which supports the previous finding that low-dose TBZC could effectively replace pharmacological doses of ZnO to alleviate diarrhea (6). The anti-diarrheal effect of TBZC may be closely related to its effects on gut health, at least in part. Sequencing results showed that the inclusion of TBZC in piglet diets could improve the diversity of intestinal microbial communities and increase the abundance of beneficial bacteria (27). Xia (24) reported that dietary TBZC supplementation increased the expression of antioxidant enzymes in the jejunum and down-regulated the expression of pro-inflammatory cytokine in the ileum, which was beneficial to improve gut barrier function. In addition, it has also been presented that 2,000 mg Zn/kg from TBZC increased the expression of the tight junction proteins and decreased intestine permeability (9). This partially hindered the translocation of harmful bacteria or their toxins, thereby reducing the risk of diarrhea and inflammation (28).

In this study, the ATTD of energy and CP in piglets from the TBZC group was higher than that from the CON group, partially explaining the growth-promoting effect of TBZC. The enhancement of nutrient digestibility may be related to the improvement of the small intestinal mucosal morphology by TBZC, thereby promoting the absorption of nutrients (24). Studies had shown that high-dose of Zn could effectively inhibit the adhesion and growth of harmful bacteria in the intestine (29, 30). This prebiotic effect could reduce the competition between microbiota and host for available starch and sugars, hence improving the efficiency of energy utilization (21). Zhang and Guo (31) reported that adding TBZC to the weaned piglet diets could promote the synthesis and secretion of pancreatic chymotrypsin, which may be one of the reasons for the increase in protein digestibility. In addition, the current study showed that dietary supplemented with 1,600 mg Zn/kg from ZnO failed to improve nutrient digestibility, but led to higher fecal Zn excretion than the TBZC group. Therefore, replacing ZnO with low dose TBZC in weaned piglet diets was beneficial to improve nutrient digestibility and abate environmental pollution to a certain extent.

The serum ALP activity could be used as a biological indicator to evaluate the status of Zn in pigs (32). It has been reported that serum ALP activity increased linearly as the dietary supplemental TBZC concentration increased (6). Cho et al. (33) proposed that increasing the absorption of Zn could directly stimulate the improvement of serum ALP activity. As observed in the present study, dietary supplementation with 1,000 mg Zn/kg from TBZC showed an increase in serum ALP activity as effective as 1,600 mg Zn/kg ZnO treatment, which may support the statement that the bioavailability of Zn in TBZC was higher than that in ZnO (6). The ALT, as a critical indictor of liver damage, leaks out into the plasma when the hepatic cells are injured (4). In both ZnO and TBZC treatment group, the decrease serum ALP activity on day 14 may be related to the alleviation of oxidative liver injure, which was caused by weaning stress (6, 23). IGF-I is a critical growth-promoting polypeptide, which is closely related to the nutritional status of animals (34). Our research showed that the levels of GH and IGF-I in the serum were unaffected by treatment with 1,600 mg Zn/kg from ZnO, which is in agreement with our previous findings (21). Carlson et al. (34) demonstrated that dietary ZnO supplementation at 1,000 mg/kg did not change the level of serum IGF-I, but at a dose of 2,500 mg/kg markedly increased the level of serum IGF-I. These results suggested that 1,600 mg/kg ZnO may not be sufficient to improve the growth performance and health status of weaned piglets. Additionally, serum GH and IGF-I levels were significantly increased in TBZC group, which was partially agreed with Zhang and Guo (25) that the levels of serum GH and Ghrelin were improved when 2,000 mg Zn/kg from TBZC was fed to piglets. Therefore, compared with traditional ZnO, TBZC can improve the growth performance of piglets with a lower dose.

The SCFA, primarily produced by microbial fermentation in the hindgut, is beneficial for enhancing gut barrier integrity and immunity regulation (35). In the current study, the concentrations of acetate, propionate, and total SCFA in the feces of piglets from TBZC treatment group were remarkably improved, which is in agree with the previous study (31). Acetate and propionate are mainly transported to the liver through the portal vein and participate in energy metabolism (35). Recent research also revealed that propionate had positive effects on the intestinal development and barrier function of pigs (36). Therefore, the improved SCFA profile in TBZC group was able to provide additional energy available to weaned piglets and may be conducive to improving gut health. Several studies found that the inclusion of 1,600–2,000 mg Zn/kg from ZnO in piglet diets significantly enhanced acetate concentration in cecal digesta or feces, which was similar to the results of the ZnO group in this study (21, 37). In addition, lactic acid concentration in the ZnO group had a tendency to decrease on d 14, which may be due to the specific inhibitory effect of high-dose ZnO on some members of the Lactobacillus genus, and this effect mainly occurred in the first 2 weeks after weaning (38).

The composition and diversity of the microbial community are important indicators of gut stability and function, and are closely related to host health. Vahjen et al. (30) reported that dietary ZnO supplementation at 3,000 mg Zn/kg increased the Richness and Shannon indices of ileal digesta. Similarly, Starke et al. (39) indicated that feeding 2,500 mg Zn/kg from ZnO to piglets can increase microbial diversity in digesta of small intestinal. In the current study, we found that the Ace and Chao indices of fecal microbes in the TBZC group were higher than those in the CON group, but supplementing 1,600 mg Zn/kg from ZnO failed to improve microbial diversity. Increased microbial diversity helped maintain gut ecosystem stability and resistance to environmental stress, thereby reducing the incidence of diarrhea (40). We also found an increasing trend in the abundance of Prevotellaceae in fecal samples by feeding different forms of Zn sources, which was similar to a previous study (14). Members of the Prevotellaceae, such as *Prevotellaceae_NK3B31_group*, could assist piglets in degrading polysaccharides in plant cell walls into SCFA, thereby providing energy for piglets and improving intestinal barrier integrity and functions (41). The family Veillonellaceae was considered as a major contributor in producing propionate in the gut (42), hence higher abundance of Veillonellaceae (22.06%) in TBZC group may partly explain the elevated propionate concentration in this treatment group. It had been reported that *Parabacteroides* played a positive regulatory role in glucose and lipid metabolism and could increase the level of succinic acid in the body (43). Koh et al. (44) found that *Parabacteroide*s can promote gut health by increasing the expression of intestinal tight junction proteins. *Succinivibrio* is a high-cellulose-degrading bacteria with the ability to degrade complex carbohydrates, which helped piglets to digest feed better (45). As a result, the increased abundance of *Parabacteroides* and *Succinivibrio* in the ZnO group was also beneficial to the health of piglets. However, the limitation of the study is that the pigs were not euthanized to collect samples of intestine digesta and tissues in all dietary treatments, therefore, the effects of TBZC on gut health of weaned piglets, including intestinal microbial community and immunological function, have not been clear. Further investigation is required to explore the role of TBZC supplementation on regulating gut health of weaned piglets.

## Conclusions

The current study demonstrated that dietary supplementation with 1,000 mg Zn/kg from TBZC had better effects on the growth performance, diarrhea alleviation, nutrient digestibility, and levels of serum growth-related hormones as compared with mg Zn/kg from ZnO supplied for weaned piglets. Additionally, TBZC supplementation improved fecal microbiota and reduced the fecal Zn excretion.

## Data Availability Statement

The raw data of DNA extract were uploaded to NCBI SRA database, with the accession number: PRJNA844880.

## Ethics Statement

The animal study was reviewed and approved by Institutional Animal Care and Use Committee of China Agricultural University.

## Author Contributions

GZ analyzed the data and drafted the manuscript. GZ, GH, and JZ performed experiments. GH and JZ conceived and designed the manuscript. GH and ZY reviewed the manuscript and given critical suggestions. JZ finalized the manuscript. All authors read and approved the final manuscript.

## Funding

This research was funded by the National Natural Science Foundation of China (31772612) and the National Swine Industrial and Technology System of China (CARS-35).

## Conflict of Interest

GZ and GH were employed by Nutrition Laboratory of Wellhope Foods Co., Ltd. The remaining authors declare that the research was conducted in the absence of any commercial or financial relationships that could be construed as a potential conflict of interest.

## Publisher's Note

All claims expressed in this article are solely those of the authors and do not necessarily represent those of their affiliated organizations, or those of the publisher, the editors and the reviewers. Any product that may be evaluated in this article, or claim that may be made by its manufacturer, is not guaranteed or endorsed by the publisher.
